# Adipose Tissue Gene Expression of Factors Related to Lipid Processing in Obesity

**DOI:** 10.1371/journal.pone.0024783

**Published:** 2011-09-22

**Authors:** Mercedes Clemente-Postigo, Maria Isabel Queipo-Ortuño, Diego Fernandez-Garcia, Ricardo Gomez-Huelgas, Francisco J. Tinahones, Fernando Cardona

**Affiliations:** 1 Laboratorio de Investigaciones Biomédicas del Hospital Virgen de la Victoria, Málaga (Fundación IMABIS), Spain; 2 CIBER Fisiopatología de la Obesidad y la Nutrición, Spain; 3 Servicio Endocrinología y Nutrición del Hospital Universitario Virgen de la Victoria, Málaga, Spain; 4 Servicio de Medicina Interna del Hospital Regional Carlos Haya, Spain; Pennington Biomedical Research Center, United States of America

## Abstract

**Background:**

Adipose tissue lipid storage and processing capacity can be a key factor for obesity-related metabolic disorders such as insulin resistance and diabetes. Lipid uptake is the first step to adipose tissue lipid storage. The aim of this study was to analyze the gene expression of factors involved in lipid uptake and processing in subcutaneous (SAT) and visceral (VAT) adipose tissue according to body mass index (BMI) and the degree of insulin resistance (IR).

**Methods and Principal Findings:**

VLDL receptor (VLDLR), lipoprotein lipase (LPL), acylation stimulating protein (ASP), LDL receptor-related protein 1 (LRP1) and fatty acid binding protein 4 (FABP4) gene expression was measured in VAT and SAT from 28 morbidly obese patients with Type 2 Diabetes Mellitus (T2DM) or high IR, 10 morbidly obese patients with low IR, 10 obese patients with low IR and 12 lean healthy controls. LPL, FABP4, LRP1 and ASP expression in VAT was higher in lean controls. In SAT, LPL and FABP4 expression were also higher in lean controls. BMI, plasma insulin levels and HOMA-IR correlated negatively with LPL expression in both VAT and SAT as well as with FABP4 expression in VAT. FABP4 gene expression in SAT correlated inversely with BMI and HOMA-IR. However, multiple regression analysis showed that BMI was the main variable contributing to LPL and FABP4 gene expression in both VAT and SAT.

**Conclusions:**

Morbidly obese patients have a lower gene expression of factors related with lipid uptake and processing in comparison with healthy lean persons.

## Introduction

The prevalence of obesity has increased over recent decades and it is now considered a major health problem [1]. Obesity is usually related to a number of co-morbidities, such as Type 2 Diabetes Mellitus (T2DM), insulin resistance (IR), cardiovascular disease or dyslipidemia, though there exist metabolically healthy obese patients without these obesity-related complications [1], [2]. It has been proposed that the expansion capacity of the adipose tissue rather than the adipose tissue size could be, at least in part, responsible for these inter-individual differences [3], and fat accumulation in the visceral adipose tissue (VAT) is a higher risk factor for the development of clinical complications of obesity than is the subcutaneous fat depot [4]. Thus, correct lipid storage and processing in the adipose tissue is crucial since if this process fails, an excess of circulating free fatty acids (FFA) will be taken up by non-adipose tissues, leading to metabolic complications such as IR and T2DM [3].

FFA entry into the adipocyte is the first step to lipid storage. The triglycerides transported in triglyceride-rich lipoproteins (TRL), i.e. chylomicrons and VLDL, are lipolyzed by lipoprotein lipase (LPL) to FFA, which are then taken up by the adipocyte. In peripheral tissues, such as adipose tissue, the rate-limiting step for triglyceride catabolism or FFA storage is catalyzed by LPL, which is bound to glycosaminoglycans at the luminal side of the capillary endothelium. LPL is the main gatekeeper enzyme for the entry and reesterification of FFA in adipose tissue [5].

In addition to LPL-catalyzed hydrolysis of triglycerides it is worth noting that TRL as well as lipase-hydrolyzed TRL remnants can also be whole-particle internalized directly by cells via LDL receptor family member proteins such as VLDL receptor (VLDLR) and LDL receptor-related protein-1 (LRP1) [6]. The VLDLR has a primary role in the anchorage of TRLs, thereby facilitating subsequent triglyceride hydrolysis by LPL and FFA entry into underlying tissues [7]. In addition, VLDLR and LPL have a reciprocal regulation since LPL enhances the binding of TRL to the VLDLR [5] and, at the same time, VLDLR regulates LPL activity, as shown in vldlr-/- mice, where LPL expression is reduced [7]. LRP1 interacts with apoE-enriched chylomicron and VLDL remnants [8] and it is stimulated by insulin in adipocytes, resulting in an increased uptake of triglycerides and cholesteryl ester from remnant lipoproteins [9]. Murine studies with adipocyte-specific inactivation of the LRP1 gene have shown that LRP1 expression in these cells plays an important role in obesity development since these mice displayed a delayed postprandial lipid clearance and were resistant to diet-induced obesity [10].

Intracellularly, FFA are bound to cytoplasmic fatty acid binding proteins (FABPs). These FABPs direct FFA into the different metabolic pathways [11]. Adipocyte fatty acid-binding protein (A-FABP or FABP4 or aP2) is a small lipid-binding protein, highly expressed in adipose tissue and also expressed in macrophages. It is one of the most abundant cytoplasmic proteins in mature adipocytes [11] and its function has been associated with insulin sensitivity, lipid metabolism and inflammation [12].

Adipose tissue secretes a number of key molecules for regulation of lipid metabolism. The acylation stimulating protein (ASP), which stimulates triglyceride synthesis, is a small protein synthesized by adipose tissue and it is the result of the posttranslational modification of the complement factor C3. ASP stimulates reesterification of FFA into triglycerides in adipocytes and fibroblasts [13], reduces endogenous FFA production by inhibiting hormone-sensitive lipase [14], and stimulates intracellular uptake of glucose by adipocytes, fibroblasts, and muscle cells [13].

The aim of this study was to analyze the mRNA expression of genes related with lipid uptake and processing in the visceral (VAT) and subcutaneous adipose tissue (SAT) of lean healthy controls, obese persons, morbidly obese patients with low IR and morbidly obese patients with high IR or T2DM to determine any differences between these phenotypes. To date, only a few human studies have been undertaken simultaneously in the two tissues, and ours is the first to compare the gene expression of this cluster of genes between these groups of patients.

## Results

The anthropometric and biochemical variables of the morbidly obese patients, the obese persons and the lean healthy controls are summarized in Table 1.

**Table 1:** Anthropometric and biochemical characteristics of the morbidly obese patients, and the obese and lean healthy subjects.

	MO HIR/T2DM(n = 28)	MO LIR (n = 10)	Obese (n = 10)	Lean (n = 12)	p
Age (years)	42.77±8.50	38.30±9.35	42.38±18.67	44.58±10.96	0.158
%male/female	25/75	30/70	25/75	41.7/58.3	0.751
Weight (Kg)	143.41±24.03	136.44±28.51	82.00±11.79	68.42±12.12	0.000
BMI (Kg/m^2^)	53.77±7.11	50.20±7.97	31.68±1.70	24.49±2.47	0.000
Waist (cm)	139.83±14.66	130.40±13.60	97.38±6.91	85.04±10.47	0.000
Hip (cm)	153.83±12.68	148.65±17.63	114.25±4.40	99.92±5.48	0.000
Ratio Waist/Hip	0.91±0.08	0.88±0.10	0.85±0.07	0.85±0.08	0.151
Insulin (µUI/mL)	24.58±12.67	12.58±3.35	9.22±2.81	7.11±2.35	0.000
Glucose (mmol/L)	6.32±1.58	4.80±0.43	5.00±0.42	4.66±0.52	0.000
Uric acid (µmol/L)	376.70±92.16	370.97±56.09	214.72±45.57	252.29±50.37	0.000
Cholesterol(mmol/L)	5.04±2.30	4.58±1.09	5.19±1.12	5.21±0.97	0.451
TG (mmol/L)	1.73±0.99	1.19±0.74	1.67±0.90	0.99±0.34	0.012
FFA (mmol/L)	0.55±0.20	0.50±0.25	0.63±0.18	0.50±0.23	0.279
HDL-C (mmol/L)	1.14±0.26	1.08±0.24	1.46±0.22	1.49±0.41	0.003
LDL-C (mmol/L)	3.26±0.95	2.51±0.52	2.79±0.87	3.14±0.61	0.157
GOT (units/L)	21.22±11.74	20.30±6.04	17.75±5.04	21.92±4.36	0.334
GPT (units/L)	43.41±20.10	47.00±9.87	40.63±10.27	42.58±10.27	0.437
GGT (units/L)	37.39±21.49	37.50±11.53	24.38±6.74	25.75±9.57	0.024
HOMA-IR	6.68±3.49	2.70±0.66	2.06±0.64	1.48±0.50	0.000
SBP (mmHg)	145.09±21.23	136.22±23.18	129.25±14.93	109.00±21.20	0.001
DBP (mmHg)	84.18±16.02	82.33±12.86	79.75±7.98	74.82±20.99	0.205
Leptin (ng/mL)	58.99±18.50	66.82±39.00	14.72±14.04	14.37±17.19	0.000
Adiponectin(ng/mL)	9.12±4.28	9.21±2.87	15.01±5.88	24.70±18.30	0.003

Values are presented as means ± s.d. p value represents statistical significance between the study groups determined by Kruskal-Wallis test. MO HIR/T2DM, morbidly obese patients with high insulin resistance or with type 2 diabetes mellitus; MO LIR, morbidly obese patients with low insulin resistance; BMI, body mass index; TG, triglycerides; FFA, free fatty acids; HDL-C, HDL cholesterol; LDL-C, LDL cholesterol; GOT, glutamic oxalacetic transaminase; GPT, glutamic pyruvate transaminase; GGT, γ–glutamyltransferase; HOMA-IR, Homeostasis Model Assessment of insulin resistance; SBP, Systolic Blood Pressure; DBP, Diastolic Blood Pressure.

The mRNA expression of the genes studied in the VAT was greater in the lean controls than in the morbidly obese patients and/or the obese patients, as summarized in Table 2. Specifically, LPL gene expression was significantly higher in lean controls than in the other study groups. FABP4 gene expression was significant higher in lean controls and in obese persons than in the two groups of morbidly obese patients. We observed that LRP1 gene expression was greater in lean controls compared to the morbidly obese groups and finally, C3/ASP gene expression was significantly higher in lean controls than in the other study groups.

**Table 2:** mRNA expression levels of the genes studied in subcutaneous adipose tissue and visceral adipose tissue in the lean healthy controls, obese subjects and morbidly obese patients.

		MO HIR/T2DM (n = 28)	MO LIR (n = 10)	Obese (n = 10)	Lean (n = 12)	p
**LPL**	VAT	2.867±2.239	3.906±2.323	3.022±1.483	6.482±2.744	0.001
	SAT	4.306±2.022*	5.841±2.932	5.975±2.053*	6.772±4.199	0.049
**FABP4**	VAT	11.996±7.349	12.583±7.868	22.241±9.134	26.078±11.712	0.001
	SAT	14.776±5.425	14.167±4.685	20.338±6.939	24.467±11.668	0.010
**ASP**	VAT	3.223±2.186	2.135±1.411	2.336±0.646	4.171±1.714	0.046
	SAT	1.337±0.995*	1.056±0. 442	1.103±0.312*	1.341±1.078**^1^**	0.929
**LRP1**	VAT	0.381±0.120	0.269±0.110	0.411±0.099	0.458±0.329	0.074
	SAT	0.498±0.228*	0.403±0.140*	0.580±0.141*	0.539±0.262	0.196
**VLDLR**	VAT	0.481±0.299	0.624±0.291	0.640±0.305	0.818±0.746	0.457
	SAT	0.572±0.272	0.629±0.294	0.848±0.264	0.959±0.969	0.198

Values are presented as means ± s.d. p value represents statistical significance between the study groups determined by Kruskal-Wallis test.

*p<0.05 between SAT and VAT. MO HIR/T2DM, morbidly obese patients with high insulin resistance or with type 2 diabetes mellitus; MO LIR, morbidly obese patients with low insulin resistance; VAT, visceral adipose tissue; SAT, subcutaneous adipose tissue. mRNA-level results are expressed as the expression ratio relative to Cyclophilin A gene expression by calculating 2^−ΔCt^, according to the manufacturer's guidelines.

In the SAT, the gene expression differences between groups were less than in the VAT, though the tendency was the same as that seen in VAT: a higher mRNA expression in lean controls than in the other study groups. Only FABP4 and LPL gene expression was significantly different between the study groups, noting a great difference between mRNA levels in lean controls and the morbidly obese groups in the SAT (Table 2).

Comparison of the gene expression levels between the VAT and the SAT in each study group showed a tendency to a higher mRNA expression in the SAT than in the VAT for all genes studied, except for C3/ASP gene expression which tended to be lower in the SAT than in the VAT. More specifically, LRP1 gene expression was significantly higher in SAT than in VAT in the obese (p = 0.018), MO LIR/T2DM (p = 0.007) and MO HIR (p = 0.007) patient groups; LPL gene expression was significantly greater in SAT than in VAT in the obese (p = 0.012) and MO HIR/T2DM (p = 0.002) groups. FABP4 gene expression was higher in SAT than in VAT (p = 0.053) in the MO HIR/T2DM group. In contrast, ASP gene expression was significantly lower in SAT than in VAT in the MO LIR/T2DM group (p = 0.000), in lean healthy controls (p = 0.003) and in the obese group (p = 0.012).

Correlations were analyzed between the mRNA expression level and BMI, plasma insulin level, HOMA-IR and plasma triglyceride levels in the VAT and the SAT (Tables 3 and 4). VAT LPL and FABP4 mRNA expression showed a significant inverse correlation with BMI, HOMA-IR and plasma insulin levels (p<0.01). LPL gene expression in the SAT also correlated inversely with BMI (p<0.01), plasma insulin levels, HOMA-IR and triglyceride levels (p<0.05). FABP4 gene expression in the SAT only correlated negatively with BMI (p<0.01) and HOMA-IR (p<0.05).

**Table 3:** Correlations between the visceral adipose tissue mRNA expression levels of the genes studied and the BMI, triglycerides, insulin and HOMA-IR in all study subjects.

	BMI	Insulin	HOMA-IR	TG
**LPL**	−0.450^a^	−0.329^a^	−0.324^a^	−0.226^b^
**FABP4**	−0.529^a^	−0.336^a^	−0.334^a^	−0.199
**LRP1**	−0.022	0.041	0.129	−0.047
**VLDLR**	−0.213	−0.162	−0.221	0.048
**ASP**	0.060	0.187	0.170	0.045

Correlations were determined by Spearman's correlation coefficient test.

a P <0.01.

b P <0.05;

BMI, Body Mass Index; HOMA-IR, Homeostasis Model Assessment of insulin resistance; TG, triglycerides.

**Table 4:** Correlations between the subcutaneous adipose tissue mRNA expression levels of the genes studied and the BMI, triglycerides, insulin and HOMA-IR in all study subjects.

	BMI	Insulin	HOMA-IR	TG
**LPL**	−0.320^a^	−0.215^b^	−0.231^b^	−0.270^b^
**FABP4**	−0.365^a^	−0.139	−0.195^b^	−0.119
**LRP1**	−0.127	−0.006	0.149	−0.025
**VLDLR**	−0.206	−0.102	−0.142	−0.077
**ASP**	0.127	0.164	0.189	0.059

Correlations were determined by Spearman's correlation coefficient test.

a P <0.01;

b P <0.05.

BMI, Body Mass Index; HOMA-IR, Homeostasis Model Assessment of insulin resistance; TG, triglycerides.

A multiple linear regression analysis was carried out to evaluate the contribution of the different variables in LPL and FABP4 gene expression in the VAT and the SAT (Tables 5 and 6). BMI, HOMA-IR, plasma insulin and triglyceride levels, age and gender were included as independent variables and LPL and FABP4 gene expression as dependent variables. The only variable that remained significantly associated with LPL and FABP4 gene expression in both the VAT and in the SAT was the BMI.

**Table 5:** Multiple regression analysis for LPL gene expression in VAT and SAT, as dependent variable, in all the study subjects.

	LPL_VAT (R = 0.434;R^2^ = 0.189)			LPL_SAT (R = 0.302; R^2^ = 0.091)			
	β	P	CI 95%	β	P	CI 95%	
BMI	−0.434	0.000	−0.120 to−0.039	−0.302	0.012	−0.106 to −0.013	
Insulin	−0.089	0.525	-	0.012	0.935	-	
HOMA-IR	−0.090	0.524	-	−0.055	0.712	-	
TG	−0.087	0.454	-	−0.198	0.105	-	

VAT, visceral adipose tissue; SAT, subcutaneous adipose tissue; CI, confidence interval; BMI, body mass index; HOMA-IR, Homeostasis Model Assessment of insulin resistance; TG, triglycerides.

**Table 6:** Multiple regression analysis for FABP4 gene expression in VAT and SAT, as dependent variable, in all the study subjects.

	FABP4_VAT (R = 0.516;R^2^ = 0.266)			FABP4_SAT (R = 0.368;R^2^ = 0.135)		
	β	P	CI 95%	β	P	CI 95%
BMI	−0.516	0.000	−0.518 to −0.218	−0.368	0.002	−0.339 to −0.079
Insulin	−0.007	0.956	-	0.124	0.392	-
HOMA-IR	−0.026	0.845	-	0.056	0.704	-
TG	−0.021	0.852	-	−0.061	0.611	-

VAT, visceral adipose tissue; SAT, subcutaneous adipose tissue; CI, confidence interval; BMI, body mass index; HOMA-IR, Homeostasis Model Assessment of insulin resistance; TG, triglycerides.

**Figure 1:**
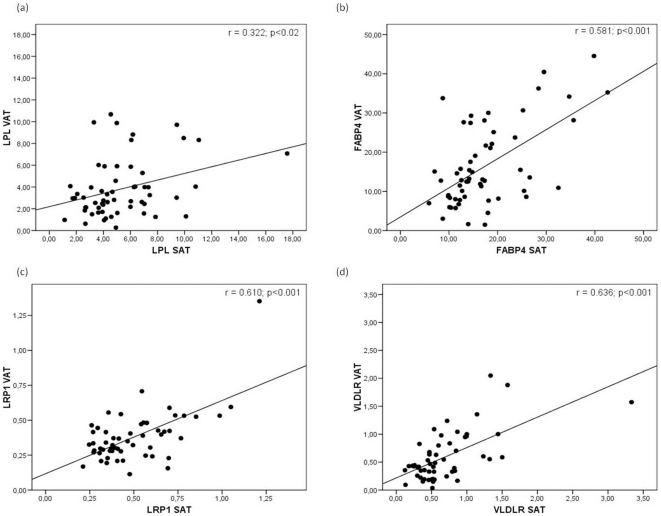
Linear relationship determined by Spearman's correlation coefficient test between (a) LPL gene expression in VAT and LPL gene expression in SAT, (b) FABP4 gene expression in VAT and FABP4 gene expression in SAT, (c) LRP1 gene expression in VAT and LRP1 gene expression in SAT and (d) VLDLR gene expression in VAT and VLDLR gene expression in SAT. VAT, visceral adipose tissue; SAT, subcutaneous adipose tissue.

Analysis was also made of the association between mRNA levels of the studied genes. Significant positive correlations were found in both VAT and SAT between the mRNA levels of the different genes: VLDLR mRNA levels correlated positively with LPL, FABP4 and LRP1 mRNA levels; FABP4 correlated positively with LRP1 and LPL; and ASP correlated positively with LRP1 (Table 7). SAT LPL gene expression correlated significantly with its corresponding VAT gene expression (Fig. 1a; p<0.02). The same was seen with FABP4 (Fig. 1b), LRP1 (Fig. 1c) and VLDLR (Fig. 1d) gene expression in VAT and SAT (p<0.001).

**Table 7:** Correlations between mRNA levels of the studied genes in both visceral and subcutaneous adipose tissue.

	LPL	FABP4	LRP1	VLDLR	ASP
**ASP**	−0.053	−0.106	0.399 a	−0.003	
**VLDLR**	0.615 a	0.419 a	0.396 a		0.119
**LRP1**	0.182	0.268 b		0.304**^2^**	0.413 a
**FABP4**	0.723 a		0.299 b	0.430 a	0.197
**LPL**		0.619 a	0.198	0.705 a	−0.039

Correlations were determined by Spearman's correlation coefficient test.

a P <0.01;

b P <0.05.

Upper table represents correlations between mRNA levels of the different genes in the VAT and the lower table represents correlations between mRNA levels of the different genes in the SAT. VAT, visceral adipose tissue; SAT, subcutaneous adipose tissue.

## Discussion

Our results indicate that the greater the degree of obesity the lower the gene expression of genes related with lipid processing in adipose tissue (LPL, FABP4, ASP and LRP1), both visceral and subcutaneous, though more obviously in the VAT.

Both LRP1 and VLDLR are members of the LDL receptor family and participate in TRL LPL hydrolysis and TRL - remnant clearance. Studies dealing with LRP1 expression in human adipose tissue are scarce. Masson et al. recently reported that LRP1 mRNA expression in human VAT is increased in obese patients with respect to lean controls [15]. In contrast, we observed a tendency for lower LRP1 gene expression in the VAT of morbidly obese patients compared to lean controls. Of note, however, is the different patient groups in the studies, since our patient group covered a greater range of degrees of obesity and included an extremely obese patient group while the study by Masson compared morbidly obese patients with a lower BMI than our patients vs. lean controls, for whom no clinical information was provided, so these data have to be considered with caution.

Animal models have shown that VLDLR is highly expressed in adipose tissue [16], but no studies have yet explored VLDLR expression in human adipose tissue, at least as far as we are aware. This, therefore, is the first study to report mRNA expression in human VAT and SAT in both lean healthy subjects and in obese patients. Studies with VLDLR knock-out mice have linked VLDLR with obesity. When vldlr ^−^/^−^ mice were fed a high-fat diet, they remained lean whilst their wild-type littermates developed obesity. Moreover, vldlr ^−^/^−^ mice experienced a reduction in adipocyte size, suggesting reduced adipocyte triglyceride storage, and their LPL activity was reduced [17], [18]. These results highlight the important role of VLDLR in TRL hydrolysis and clearance and triglyceride storage in adipose tissue. Concordantly, in humans VLDLR deficiency has been reported and affected patients were also underweight [19]. In addition, VLDLR variants have been associated with a lower BMI [20], but no research has been done on how this mutation can affect VLDLR expression or function. In our study we did observe a tendency towards a lower VLDLR gene expression as the BMI increased. Studies of human VLDLR regulation are few [16] and we are conscious of the limitations in our study deducing a mechanistic hypothesis. Nonetheless, our study represents a first approach in the study of VLDLR in human adipose tissue and further studies will be necessary to understand the relationship between VLDLR in human adipose tissue, obesity and insulin resistance.

Intracellularly, FABPs are believed to sequester and direct FFA and their derivatives to different metabolic pathways, thus playing an important role in fatty acid metabolism and avoiding intracellular lipotoxicity [7]. Several studies have shown a higher gene expression of FABP4 in the SAT than in the VAT of obese subjects [21], [22]. Concordantly, we observed the same tendency in morbidly obese patients. However, other authors failed to find a significant difference between FABP4 gene expression in SAT and VAT [23]. We found that FABP4 gene expression was significantly lower in the two morbidly obese groups than in obese and lean subjects in the VAT and in the SAT. In a previous study with obese normoglycemic, obese glucose intolerant, obese diabetic patients and lean controls, a significant difference was only found in the SAT between normoglycemic obese and glucose intolerant obese female patients [23]. However, we found no differences between the groups according to the degree of IR and multiple regression analysis showed that BMI was the only variable explaining the expression levels of FABP4, in both VAT and SAT. It is worth noting that the study of Poulain-Godefroy et al. was carried out in just women with a lower BMI than our morbidly obese patients, so it is likely that the BMI effect on FABP4 mRNA expression is only noticeable in patients with a very high BMI. Our group has previously shown that gene expression of FABP4 was lower in morbidly obese persons than in lean persons in SAT [24]. In the present study was also included a group of non morbidly obese persons with a low IR and showed that in this group of patients with a lower BMI, the expression levels of FABP4 were more similar to the group of healthy lean persons.

When there is excessive supply of nutrients, the adipose tissue fatty acids are reesterified in triglycerides to be stored in adipose tissue. ASP has been shown to be a powerful activator of this process in adipocytes, mainly due to a greater activity of diacylglycerol acyltransferase (DGAT), to greater entry of glucose into the cell (necessary for glycerol synthesis) via greater externalization of the GLUT receptors, and finally, inhibition of hormone sensitive lipase (HSL) [13], [14]. In addition, ASP indirectly increases LPL activity [25]. Plasma ASP levels are increased in obesity [13]. However, obesity is not an essential feature of elevated plasma ASP levels, as ASP is increased in subjects with diabetes or polycystic ovary syndrome, even in the absence of obesity [26]. But, since ASP has an autocrine effect in adipose tissue, the use of plasma ASP levels is not the best method to estimate adipose ASP production and its metabolic effect and the *in situ* measurement of ASP production would have a greater biological significance. ASP is produced in the adipose tissue by the posttranslational cleavage of C3 complement factor [13]. Few studies have examined C3 gene expression in adipose tissue [27]–[31]. Our study is the first to compare the ASP precursor (C3) gene expression in SAT and VAT from morbidly obese patients with low IR and with high IR or T2DM, obese patients with low IR and lean healthy subjects. Koistinen et al. found that overweight patients, with and without T2DM, had a higher C3 gene expression than lean healthy controls in the SAT [29]. However, we found no significant differences between our study groups in SAT C3 gene expression, but lean controls had higher VAT ASP mRNA levels than the obese groups. We also saw that ASP gene expression was greater in VAT than SAT, in agreement with earlier studies [28], [30]. VAT has a lower response capacity to ASP and it has been proposed that the higher expression levels of its precursor may be a compensatory mechanism against its low response capacity [28], [30].

LPL plays a major role in lipid metabolism and transport. The availability of VLDL-derived fatty acids for energy needs and adipocyte storage is dependent upon hydrolytic activity of LPL, and to a lesser extent, endothelial lipase [5]. Insulin has a major effect on LPL regulation in adipose tissue since in mature adipocytes insulin not only increases the level of LPL mRNA but also regulates LPL activity through both posttranscriptional and posttranslational mechanisms [5], [32]. Glucose also increases adipose tissue LPL activity but, unlike insulin, glucose does not affect the level of LPL mRNA [5], [33]. In our study the patients who had high levels of IR or T2DM only experienced a slight reduction in LPL expression in comparison with the other groups and the BMI was the main variable associated with LPL mRNA levels, inversely. Thus, we think the cause of this reduction in LPL expression is more probably the increase in BMI and the hypertrophy or hyperplasia of the adipose tissue rather than the degree of IR or the presence of T2DM. Moreover, there are marked variations in the activity of LPL in adipose tissue depots in humans. The steady-state mRNA levels for LPL, as well as LPL mass, are lower in VAT than SAT. Insulin increases the levels of LPL mRNA and LPL activity in abdominal SAT but not VAT [34], [35]. In our study we noted the same results. This could be because the specific LPL activity is greater in VAT than in SAT [36].

Thus, in our study subjects, as the BMI rose there was a drop in mRNA expression of the genes studied, particularly LPL and FABP4. This suggests that as the BMI increases the lipid storage capacity in adipose tissue could decrease, which could be related with the development of the IR that later appeared in these patients, as proposed by others [37]. Accordingly, our results support the following hypothesis: Under normal conditions, in the presence of excess energy the adipose tissue is entrusted with channeling this excess and storing the nutrients, at the expense of increasing its volume. In the obese state, the adipose tissue is submitted to a continuous energy surplus, such that over time the lipid storage capacity of this tissue can be surpassed and the adipose tissue, enable to respond to this excess, could reduce the expression of genes involved in lipid processing and storage. If this occurs, the adipose tissue might begin working incorrectly, leading over time to metabolic disorders. Therefore, our study shows that adipose tissue gene expression is different in subjects with different BMI which indicates a different transcriptional regulation. Nevertheless, further studies analyzing the activity and concentrations of these molecules will be necessary to confirm this hypothesis.

In conclusion, our results show that morbidly obese patients have a lower gene expression of factors related with lipid uptake and processing in comparison with healthy lean persons, and that this reduction is not dependent on the degree of insulin resistance.

## Materials and Methods

### Subjects and study design

The study was undertaken in 38 severely morbidly obese persons (BMI = 52.83±7.41 kg/m^2^), 10 obese patients (BMI = 31.68±1.70 kg/m^2^) and 12 healthy lean controls (BMI = 24.49±2.47 kg/m^2^). Patients were excluded if they had cardiovascular disease, arthritis, acute inflammatory disease, infectious disease, renal disease or were receiving drugs that could alter the lipid profile or the metabolic parameters at the time of inclusion in the study. All participants gave their informed written consent and the study was reviewed and approved by the Ethics and Research Committee.

The morbidly obese patients were divided in two groups: 10 patients with neither impaired glucose tolerance nor T2DM and low insulin resistance [homeostasis model assessment of insulin resistance (HOMA-IR) <4.7] (MO LIR), and 28 patients with high insulin resistance or T2DM (MO HIR/T2DM). The cut-off point for the HOMA-IR was taken from previous studies carried out in a healthy population with no carbohydrate metabolism disorders [38].

### Laboratory measurements

Before surgery and after an overnight fast, blood samples were obtained from the antecubital vein and placed in vacutainer tubes (BD vacutainer™, London, UK). The serum was separated by centrifugation for 10 min at 4000 rpm and immediately frozen at −80°C until analysis. Serum glucose, cholesterol, triglycerides and HDL cholesterol were measured in a Dimension autoanalyzer (Dade Behring Inc., Deerfield, IL) by enzymatic methods (Randox Laboratories Ltd., UK). The LDL cholesterol was calculated from the Friedewald equation. Insulin was quantified by radioimmunoassay supplied by BioSource International, Camarillo, S.A. Leptin and adiponectin were analyzed by enzyme immunoassay (ELISA) kits (respectively: DSL, Webster, TX, and DRG Diagnostics GmbH, Germany). The homeostasis model assessment of insulin resistance (HOMA-IR) was calculated from fasting insulin and glucose with the following equation: HOMA-IR = fasting insulin (µIU/mL)×fasting glucose (mmol/L)/22.5 [39].

### Visceral and subcutaneous adipose tissue RNA isolation and real-time quantitative PCR

VAT and abdominal SAT were obtained during bariatric surgery in the morbidly obese patients or during hiatal hernia surgery in lean healthy controls and obese patients. The biopsy samples were washed in physiological saline and immediately frozen in liquid nitrogen. Biopsy samples were maintained at −80°C until analysis.

Total RNA isolation from adipose tissues was obtained using RNeasy Lipid Tissue Mini Kit (Qiagen GmbH, Germany). The purity of the RNA was determined by the 260/280 absorbance ratio on the Nanodrop. The integrity of total purified RNA was checked by denaturing agarose gel electrophoresis and ethidium bromide staining. For first-strand cDNA synthesis, a constant amount of 1 µg of total RNA was reverse transcribed using random hexamers as primers and Transcriptor Reverse Transcriptase (Roche, Mannheim, Germany). Gene expression was assessed by real-time PCR using an Applied Biosystems 7500 Fast Real-Time PCR System (Applied Biosystems, Darmstadt, Germany) with TaqMan technology. The reaction was performed, following the manufacturer's protocol, in a final volume of 25 µl. The cycle program consisted of an initial denaturing of 10 min at 95 °C, then 40 cycles of 15 sec denaturing phase at 95°C, and 1 min annealing and extension phase at 60°C. The commercially available and prevalidated TaqMan primer/probe sets used in human samples were as follows: Cyclophilin A (4326316E, RefSeq. NM_021130.3) used as endogenous control for the target gene in each reaction [40], LPL (Hs00173425_m1, RefSeq. NM_000237.2), FABP4 (Hs01086177_m1, Refseq. NM_001442.2), LRP1 (Hs00233899_m1, Refseq. NM_002332.2), VLDLR (Hs01045922_m1, Refseq. NM_003383.3) and C3/ASP (Hs00163811_m1, Refseq. NM_000064.2). A threshold cycle (Ct value) was obtained for each amplification curve and a ΔCt value was first calculated by subtracting the Ct value for human Cyclophilin A cDNA from the Ct value for each sample and transcript. Fold changes compared with the endogenous control were then determined by calculating 2^−ΔCt^, and expression results are expressed as the expression ratio relative to Cyclophilin A gene expression for humans, according to the manufacturer's guidelines. Inter-assay variability (coefficient of variation) of the housekeeping gene was less than 0.5%. All samples were quantified in triplicate and positive and negative controls were included in all the reactions.

### Statistical analysis

The results are given as the mean ± SD. Comparisons of the anthropometric and biochemical characteristics as well as the mRNA expression levels between the study groups were made with a non-parametric test (Kruskal–Wallis). Differences in mRNA expression levels between the SAT and VAT were analyzed by the Wilcoxon test. The Spearman correlation coefficients were calculated to evaluate the association between the study variables. Values were considered to be statistically significant when P<0.05. The analyses were performed with SPSS (Version 15.0 for Windows; SPSS Iberica, Madrid, Spain).
